# Authors' reply

**DOI:** 10.4103/0301-4738.55143

**Published:** 2009

**Authors:** Gowri J Murthy, Praveen R Murthy, Krishna R Murthy, Vinay V Kulkarni, K R Murthy

**Affiliations:** Glaucoma Service, Prabha Eye Clinic and Research Centre and Vittala International Institute of Ophthalmology, Bangalore, India

Dear Editor,

The authors wish to thank Raizada *et al*, for their interest in our article[1] and for their comments.[2] We would like to clarify a few points which were raised in the letter.

We admit that best corrected visual acuity (BCVA) comparisons across modalities have limitations. Additional procedures which were performed in our series, may have had an impact on BCVA, and might favor our results. However, in this subset of refractory glaucomas traditionally associated with poor visual prognosis, we have tried to demonstrate that endoscopic cyclophotocoagulation (ECP) along with addressing some of the existing comorbidities, can lead to good visual outcomes.

In our study series we have combined other procedures with ECP. However, the contribution of these procedures to intraocular pressure (IOP) lowering in our subset is very minimal, as none of our study eyes had raised IOP directly related to either silicone oil (such as pupil block, emulsified oil), or to the intraocular lens (IOL) (IOL-related pupil block). Removal of oil/explantation of IOL is unlikely to have contributed in a significant manner to the IOP reduction. Endolaser to the peripheral retina was specifically done in eyes with neovascular glaucoma, and this definitely addresses the neovascularization and allows for regression of new vessels, but cannot be adequate for management of significantly raised IOPs.

In our study phakic eyes underwent anterior ECP. We have not had any instances of lens touch by the probe, in our experience. Limbal ECP is a well-recognized modality of performing ECP, as reported in the literature.[3]

Any vitrectomy done by the pars plana route is termed as pars plana (PP) vitrectomy, in our series. Three ports have been made because we do not use irrigating light pipes. It is a standard recommendation to perform adequate vitrectomy, along with PP ECP to avoid further retinal detachments/tears.[3]
